# Management of Childhood Obesity

**DOI:** 10.3390/ijms27083528

**Published:** 2026-04-15

**Authors:** Hoda Gad, Hajar Dauleh, Idris Mohammed, Rayaz A. Malik, Khalid Hussain

**Affiliations:** 1 Lunenfeld-Tanenbaum Research Institute, Mount Sinai Hospital, Toronto, ON M5T 3H7, Canada; hoda.yahia89@gmail.com; 2 Endocrinology Department, Sidra Medicine, Doha P.O. Box 26999, Qatar; hdauleh1@sidra.org (H.D.); imohammed1@sidra.org (I.M.); 3 Department of Medicine, Weill Cornell Medicine-Qatar, Doha P.O. Box 24144, Qatar; ram2045@qatar-med.cornell.edu; 4 Institute of Cardiovascular Medicine, University of Manchester, Manchester M13 9PL, UK

**Keywords:** childhood, obesity, management

## Abstract

Obesity is characterized by an excess accumulation of adipose tissue, independent of its distribution or function, and is associated with organ dysfunction and a broad range of metabolic and cardiac complications. Nutritional, environmental, behavioral, genetic, psychological, and metabolic factors contribute to obesity. It is associated with many secondary complications, including diabetes mellitus, hypertension, dyslipidemia, metabolic dysfunction-associated steatotic liver disease, reduced quality of life, and psychological illnesses. For practical purposes, childhood obesity can be classified into polygenic, monogenic, syndromic, and endocrine causes. Management should be patient-centered and multidisciplinary, integrating lifestyle/behavioral changes with adjunctive pharmacotherapy and bariatric surgery where appropriate. In monogenic or syndromic obesity, knowing the underlying genetic etiology allows precision-guided and more effective therapy. This review aims to provide a state-of-the-art review on the management of obesity in children.

## 1. Introduction

Childhood obesity is a complex multifactorial disease due to lifestyle, nutritional, social, metabolic, environmental, and genetic factors [1]. In the US, the prevalence of obesity in children aged 12–17 years increased from 5% in 1976–1980 to 19.7% in 2017–2020 [2], meaning that around 19.7% (around 14.7 million) of children and adolescents aged 2–19 years were living with obesity in 2020. The World Obesity Federation estimated that the global prevalence of obesity in children and adolescents aged 5–19 years will increase to 254 million by 2030 [3]. A recent meta-analysis of 2033 studies reported a 1.5-fold increase in the prevalence of childhood obesity between 2012 and 2023, and it was significantly higher in children aged 6–12 years, males, those who attended private school, and those with higher maternal weight and birth weight [4]. Recent global forecasting data project a continued rise in pediatric obesity, with an estimated 1.6% of children aged 5–14 expected to have obesity by 2050 if current trends persist, highlighting a substantial and growing public health burden worldwide [5].

Childhood obesity is associated with insulin resistance [6], type 2 diabetes [7] metabolic dysfunction-associated steatotic liver disease (MASLD) [6], dyslipidemia [7], polycystic ovary syndrome [6], hypertension [7], reduced quality of life [8], poorer educational attainment [9], orthopedic problems, OSA (obstructive sleep apnea) [6], and impaired psychological health [10]. Polygenic obesity is the result of an interaction between lifestyle, environment, and multiple genetic variants [11,12]. Monogenic obesity is characterized by severe early-onset obesity due to pathogenic variants in a single gene, and syndromic obesity is usually associated with dysmorphic features and developmental delay [13], e.g., Prader–Willi syndrome (PWS) and Bardet–Biedl syndrome (BBS).

Obesity has been defined as excess adiposity, regardless of fat distribution or function, with organ dysfunction and complications like diabetes, hypertension, and fatty liver [14]. Preclinical obesity is characterized by excess adiposity without organ dysfunction but with increased risk of progression to clinical obesity, type 2 diabetes, cardiovascular disease, cancer, and mental health disorders. This narrative review provides an overview of the current evidence base and summarizes the most recent clinical guidelines related to the diagnosis and management of childhood obesity.

### 1.1. Proposed Diagnostic Framework for Pediatric Obesity

**Figure 1:**
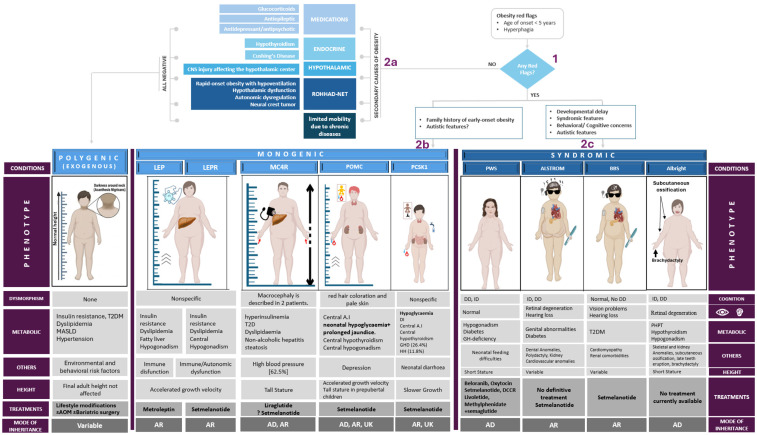
Diagnostic approach and phenotypic spectrum of pediatric obesity. Evaluation begins with screening for red flags (age of onset <5 years and hyperphagia), which should prompt consideration of pathogenic causes other than polygenic obesity, including monogenic and syndromic forms. Secondary causes of obesity—such as medication-induced, endocrine, and hypothalamic disorders, as well as conditions associated with limited mobility—should also be excluded. The diagnostic pathway is organized into sequential steps: (1) initial evaluation for red flags, followed by (2a) assessment for secondary causes of obesity, (2b) evaluation for monogenic obesity, and (2c) evaluation for syndromic obesity. The lower panel summarizes key clinical characteristics, metabolic complications, and treatment approaches across the major categories of pediatric obesity. Polygenic obesity represents the most common form and is associated with environmental and behavioral risk factors. Monogenic obesity includes defects in the leptin–melanocortin pathway (LEP, LEPR, MC4R, POMC, and PCSK1), while syndromic obesity occurs in genetic disorders such as Prader–Willi syndrome (PWS), Alström syndrome, Bardet–Biedl syndrome (BBS), and Albright hereditary osteodystrophy. Abbreviations: LEP, leptin gene deficiency; LEPR, leptin receptor gene deficiency; MC4R, melanocortin 4 receptor deficiency; POMC, proopiomelanocortin deficiency; PCSK1, proprotein convertase subtilisin/kexin type 1 deficiency; PWS, Prader–Willi syndrome; BBS, Bardet–Biedl syndrome; T2DM, type 2 diabetes mellitus; AI, adrenal insufficiency; DI, diabetes insipidus; GHD, growth hormone deficiency; HH, hypogonadotropic hypogonadism; DD, developmental delay; PHPT, pseudohypoparathyroidism; AR, autosomal recessive; AD, autosomal dominant; UK, unknown inheritance; and AOM, anti-obesity medications.

Our approach to pediatric obesity is stepwise and phenotype-driven. The initial evaluation focuses on excluding secondary causes of obesity, including endocrine disorders, hypothalamic injury, medication effects, and chronic disease-related immobility. In the absence of identifiable secondary causes and clinical red flags (age below 5 years and hyperphagia), obesity is classified as polygenic (simple/exogenous) obesity. When red flags such as early-onset obesity and hyperphagia are present, further phenotypic stratification is undertaken: the presence of developmental delay, communication disorders, learning difficulties, or syndromic features suggests a syndromic form of obesity, whereas their absence raises suspicion for monogenic obesity, warranting targeted genetic evaluation (Figure 1).

### 1.2. Polygenic Obesity

Polygenic obesity is due to the interaction of lifestyle, genetic, epigenetic, and environmental factors [15]. Polygenic obesity is influenced by hundreds of genetic variants that each confer modest risk. Genome-wide association studies (GWAS) and multi-omics approaches have revealed a complex interplay of molecular mechanisms across neural, endocrine, and metabolic systems that collectively govern energy balance and adiposity. Recent integrative studies highlight the dominant role of regulatory variation in central nervous system tissues and peripheral metabolic pathways in shaping obesity susceptibility [16].

Large-scale GWAS have identified hundreds of loci associated with body mass index (BMI) and adiposity traits. Increasing sample sizes and multi-ancestry meta-analyses are expanding locus discovery, while integrative analyses incorporating transcriptomic and epigenomic data are beginning to link variants to biological function [16]. A recent multi-omics study combining GWAS, transcriptome-wide, and epigenome-wide datasets identified 195 BMI-associated genes, with 21 prioritized through network-based analyses in adipose tissue and several overlapping signals in brain tissue, highlighting cross-tissue genetic architecture [17]. Despite this progress, variant-to-function (V2F) translation remains a major challenge: most risk variants lie in non-coding regions influencing gene expression in cell-type-specific regulatory contexts [18].

Multiple independent genetic loci have been associated with changes in BMI and body composition in children and adolescents [19,20,21]. An obesogenic environment triggers obesity-predisposing genes and epigenetic changes, resulting in obesity [22]. Indeed, twin studies have shown that the genetic inheritance of obesity is as high as 47–90% [23]. Unique independent variants (such as *MC4R*, *BDNF*, *SH2B1*, *POMC*, *LEP*, *LEPR*, *NPY*, *SIM1*, *NTRK2* and *PCSK1* [24,25] and copy number variants (CNVs) in 11q11 (*OR4P4*, *OR4S2*, and *OR4C6*), 1p21.1 (*AMY1*), 10q11.11 (*NPY4R*), 10q26.3 (*CYP2E1*), 16p11.2, 16q12.2 (*FTO*), 16p12.3. (*GPRC5b*) and 4q25 loci) [26,27] have been identified through genome-wide association studies (GWAS) in children with obesity.

A central theme in polygenic obesity is the enrichment of risk variants in regulatory regions active in neurons and in hypothalamic circuits that control appetite and energy homeostasis. Recent studies confirm that genes near BMI-associated loci are disproportionately expressed in brain tissue, especially in regions implicated in satiety and reward processing [28]. Key mechanisms include altered sensitivity to adiposity signals (e.g., leptin, insulin) at the level of hypothalamic nuclei, changes in neuropeptide expression, synaptic remodeling that biases feeding behavior, and disruption of reward circuitry (e.g., dopaminergic pathways), thereby promoting hedonic feeding.

Emerging evidence from imaging genetics suggests that higher adiposity has causal effects on brain structure, particularly cortical regions associated with reward and executive control [29]. Adipose tissue is not merely a passive storage depot but an active endocrine organ whose cellular composition and transcriptional programs are modulated by genetic risk. Polygenic obesity variants influence adipogenesis, lipid droplet dynamics, inflammatory signaling, and adipokine secretion. Recent integrative genomics has illuminated several genes acting in adipose tissue that also intersect with central regulatory pathways, underscoring cross-tissue communication in energy balance [17].

Polygenic risk interacts with environmental exposures across the lifespan. Epigenetic modifications—in response to diet, physical activity, and early life nutritional status—can shape gene expression in key metabolic tissues, potentially amplifying genetic susceptibility. Stable chromatin changes within adipocytes or neuronal circuits may underlie persistent obesogenic phenotypes and contribute to the difficulty of sustained weight loss (an “epigenetic memory”). Polygenic risk score (PRS) combines the effects of multiple genetic variants identified through GWAS by summarizing the number of risk alleles carried by an individual, weighted by their respective effect sizes, to estimate genetic susceptibility to a disease [30]. PRS shows promise for early identification of individuals at elevated obesity risk, enabling preventive interventions before significant weight gain. However, PRS performance varies by ancestry and requires further refinement for global applicability. Therapeutic targets arising from mechanistic understanding include pathways in neural appetite control, adipose tissue function, and metabolic efficiency. Gene therapy and targeted pharmacologic modulation of specific molecular nodes represent emerging areas of investigation, though clinical translation remains nascent.

Polygenic obesity emerges from distributed perturbations across neural, endocrine, and metabolic systems. Regulatory genetic variants shape tissue-specific gene expression, particularly in the brain, but also in adipose and other peripheral tissues. Integrative genomics is beginning to bridge the gap between association signals and biological mechanisms, pointing toward precision approaches for prediction, prevention, and treatment. Ongoing challenges include expanding functional annotation, enhancing multi-ancestry representation, and elucidating gene–environment interplay.

Children acquire behaviors by modeling parents and peers; thus, exposure to healthy food is a key [31]. Eating out and eating while watching TV are associated with reduced physical activity and increased exposure to advertised products, such as sweetened beverages and snacks [31,32]. Additionally, authoritative feeding without providing a rationale for children is associated with increased desire for unhealthy food [33]. Whilst fast food is undoubtedly related to obesity, it is hard to establish a causal relationship [34]. Snacks like chips and candy are linked to increased caloric intake, but the link between snacking and obesity is hard to establish [35]. In a national nutrition survey in New Zealand, children aged 5–14 years who skipped breakfast tended to have a higher BMI, presumably due to snacking during the day [36]. Furthermore, sugary drinks are less filling and consumed in larger quantities, resulting in higher caloric intake [35].

A key component of polygenic obesity is physical inactivity, and indeed, a sedentary lifestyle and increased screentime are linked to obesity [35]. Each additional hour of TV watching increases the prevalence of obesity by 2% [35], and the number of hours spent watching TV correlates with the consumption of advertised food such as sweetened cereals, sweets, sugary drinks, and salty snacks [32]. Reliance on being driven to school is associated with a higher BMI [35], while increased physical activity is associated with a lower BMI, waist circumference, and body fat percent [37].

A systematic review showed 264% increased odds of childhood obesity in children of mothers with overweight and obesity [38]. Additionally, a higher BMI during pregnancy was linked to obesity in children not only through shared genetic predisposition, but also by exposure to the obesogenic environment, surroundings that promote excessive energy intake and reduced physical activity [39]. In a randomized controlled trial, infants of mothers who remained obese post-partum were more obese than those with a normal BMI [40].

### 1.3. Endocrine-Disrupting Chemicals and Obesity

The development of obesity is increasingly linked to the widespread impact of endocrine-disrupting chemicals (EDCs), especially a subgroup called “obesogens”. These external agents, found in consumer products such as polycarbonate plastics (Bisphenol A), food can linings, and personal care items containing phthalates, interfere with the body’s hormonal regulatory systems. By mimicking or blocking natural hormones, EDCs disrupt the signaling pathways that control metabolism, appetite, and fat cell formation [41,42]. A key mechanism involves activating the peroxisome proliferator-activated receptor gamma (PPAR), the master regulator of adipocyte differentiation. This activation not only increases the number and size of fat cells (hyperplasia and hypertrophy) but also alters the “set point” for energy balance, predisposing individuals to gain weight even without excessive caloric intake [43].

The role of EDCs in the development of obesity is most important during the prenatal and early postnatal stages, when they cause lasting epigenetic changes. Exposure to phthalates and polyfluoroalkyl substances (PFAS) during these sensitive periods can reprogram metabolic pathways, leading to altered insulin sensitivity and disrupted satiety signals, such as leptin resistance [4]. These changes create a biological vulnerability to obesity that lasts throughout a person’s life and can be passed down through generations. As a result, the worldwide increase in obesity cannot be fully explained by genetics or lifestyle alone; instead, it reflects a complex interaction between environmental chemical exposure and the endocrine regulation of fat storage and energy expenditure [41,44].

### 1.4. Eating Disorders and Childhood Obesity

The coexistence of binge eating disorder (BED) and childhood obesity is a significant comorbidity that requires regular clinical attention. Studies indicate that about 26% of overweight or obese youth experience loss of control while eating, while the prevalence of full-syndrome BED in pediatric populations ranges from 1.3% to 5% [45]. These results are consistent with earlier research showing rates between 9% and 30%, with variations depending on assessment methods and population differences. The relationship appears bidirectional: binge eating behaviors are associated with faster BMI increases, and obesity itself raises the likelihood of developing disordered eating patterns [46].

This high co-occurrence has important clinical implications, as youth with obesity who engage in binge eating tend to experience more severe psychological issues, such as higher rates of mood disorders and lower quality of life [47]. Although up to 22% of surveyed youth meet criteria for some eating disorders, with those having higher BMIs being at greater risk, there are still significant gaps in screening during clinical practice. The American Academy of Pediatrics now recommends comprehensive eating disorder screening before starting obesity treatment, highlighting that early detection of BED is crucial for properly sequencing comorbid treatments in specialized care settings [48,49,50].

### 1.5. Monogenic Obesity

Monogenic obesity is characterized by early-onset obesity, “age below 5-year-old” due to pathogenic variant/s in a single gene. Most monogenic obesity genes regulate the hypothalamic leptin–melanocortin pathway, which plays a significant role in the regulation of feeding behavior and energy homeostasis [51,52,53]. Monogenic obesity accounts for approximately 5% of cases of severe obesity [54,55] due to variants in *MC4R*, *LEP*, *LEPR*, *POMC*, *PCSK1*, *PCSK2*, *SIM1*, *MC3R*, *MRAP2*, *MC3R*, *BDNF*, *SH2B1*, *NTRK2*, *DYRK1B*, and *SIM1*, and the mode of inheritance can be autosomal dominant or recessive. Pathogenic variants (homozygous and heterozygous) in the *MC4R* gene are the most frequent cause of monogenic obesity, with over 200 variants reported in the literature [56].

While most of the obesity risk arises from polygenic variation modified by environment, monogenic obesity represents rare, high-penetrance disorders caused by mutations in single genes that disrupt central energy homeostasis pathways. Patients with severe early-onset obesity offer unique insights into the neurobiological control of body weight in humans and have shaped current conceptual models of energy balance regulation [57].

The foundational discovery in monogenic obesity was the identification of congenital leptin deficiency, which highlighted the existence of endocrine feedback between adipose tissue and the brain. Leptin, a hormone secreted by adipose mass, acts on its receptor (LEPR) in the arcuate nucleus of the hypothalamus, where it modulates two primary neuronal populations: anorexigenic pro-opiomelanocortin (POMC)/CART neurons and orexigenic agouti-related peptide (AgRP)/neuropeptide Y neurons. Activation of POMC neurons increases production of melanocortin peptides (notably α-MSH), which stimulate melanocortin-4 receptors (MC4R) on second-order neurons (e.g., in the paraventricular nucleus), leading to suppression of food intake and increased energy expenditure. Conversely, AgRP antagonizes MC4R, promoting feeding behaviors [57].

This leptin–melanocortin signaling pathway constitutes the principal biological mechanism whose disruption causes monogenic forms of obesity. Mutations at different nodes of this axis result in similar clinical phenotypes, most notably early-onset hyperphagia and severe weight gain, demonstrating that proper interpretation of adiposity signals by the central nervous system is essential for energy balance.

Mutations in the leptin gene (*LEP*) lead to congenital leptin deficiency, characterized by rapid weight gain, intense hyperphagia, hypogonadotropic hypogonadism, and, in some cases, immune dysfunction due to leptin’s role in immune regulation. Homozygous frameshift and nonsense mutations abolish leptin secretion, depriving the hypothalamus of a key adiposity signal; recombinant leptin treatment in affected individuals markedly reduces appetite and normalizes weight trajectories, uniquely validating the causal mechanism in humans [58].

Leptin receptor (*LEPR*) mutations cause a phenotype comparable to ligand deficiency but result from impaired signal transduction downstream of leptin binding. LEPR mutations interfere with JAK–STAT pathway activation and cellular responses to leptin, effectively producing a genetically determined leptin-resistant state. Both *LEP* and *LEPR* deficiencies are inherited in an autosomal recessive manner and are rare worldwide, with higher prevalence in consanguineous populations.

Downstream of leptin and LEPR activation, *POMC* encodes a precursor polypeptide that is processed by proprotein convertases such as *PCSK1* (encoding PC1/3) to generate active melanocortin peptides, including α-MSH. POMC deficiency results in the absence of these melanocortin agonists, abolishing MC4R stimulation despite intact upstream signaling. Clinically, *POMC* deficiency is associated with adrenal insufficiency (ACTH deficiency) as well as severe obesity and hyperphagia, reflecting the gene’s pleiotropic roles [57].

Mutations in *PCSK1* impair prohormone processing, reducing α-MSH production among other effects, and produce a syndromic form of obesity with additional endocrinopathies. Both POMC and PCSK1 deficiencies underscore the importance of peptide maturation in maintaining normal body weight regulation.

Among monogenic causes of obesity, mutations in *MC4R* are the most frequently identified, particularly in patients with severe early-onset obesity. Heterozygous mutations are often sufficient to induce a dominant phenotype, although homozygous or compound heterozygous mutations are associated with even more severe obesity. Mutations can impair receptor folding, trafficking to the cell surface, ligand binding, or coupling to Gs proteins and downstream cAMP signaling, thereby diminishing the anorexigenic output of the pathway.

The high prevalence of *MC4R* mutations in multiple cohorts confirms its central role in human energy homeostasis, and functional studies demonstrate that even partial loss of MC4R signaling produces marked increases in body weight. Recent cohort sequencing efforts continue to identify *MC4R* as the most common genetic cause of monogenic obesity in diverse populations.

While the classical leptin–melanocortin genes account for many monogenic cases, broader genetic screening studies have expanded the landscape. Variants in genes such as *SH2B1*, *SIM1*, *BDNF*, *NTRK2*, and others have been associated with severe obesity, implicating additional pathways including synaptic plasticity, energy sensing, and neurodevelopmental processes. These discoveries reflect that hypothalamic circuitry integrity and peripheral signaling integration both contribute to body weight regulation.

The common underlying genetic variants, phenotypic features, and treatment of the various forms of monogenic childhood obesity are summarized in Table 1.

### 1.6. Syndromic Obesity

**Figure 2:**
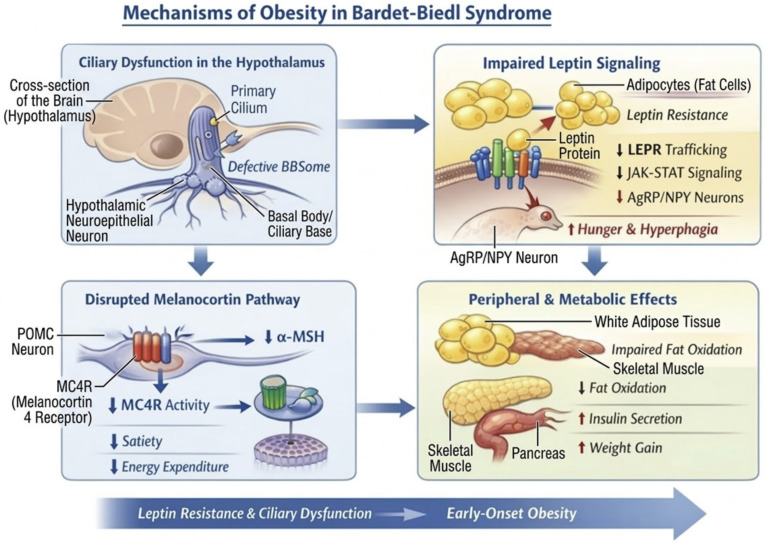
Showing the molecular mechanisms of obesity in BBS. BBSome: Bardet–Biedl syndrome protein complex; LEPR: Leptin receptor; JAK–STAT: Janus Kinase–signal transducer and activator of transcription; AgRP: Agouti-related peptide; NPY: Neuropeptide Y; POMC—Proopiomelanocortin; α-MSH—Alpha–Melanocyte-stimulating hormone; MC4R: Melanocortin-4 Receptor; ↑ Increased; ↓ Decreased.

Syndromic obesity is associated with dysmorphic features and developmental delay [13], typically due to chromosomal abnormalities with large deletions or insertions, and in some cases, single gene pathogenic variants (Table 2) [77]. At a molecular level, syndromic obesity represents a neurodevelopmental and neuroendocrine disorder in which disrupted brain circuits, impaired hormone signaling, and altered metabolism converge to produce severe early-onset obesity. Across the syndromic forms of obesity, the underlying molecular mechanisms may involve defects in hypothalamic circuits leading to misinterpretation of energy stores, which is perceived as starvation and leads to hyperphagia, and the orexigenic pathways (AgRP/NPY) may become overactive, with impaired anorexigenic pathways (POMC/CART, MC4R), and in some cases there is endocrine dysfunction (affecting growth and the adrenal and gonadal axis). Syndromes such as Alström or Bardet–Biedl involve ciliary or mitochondrial dysfunction, impairing energy sensing and fat oxidation and contributing to obesity. Bardet–Biedl syndrome (BBS) represents a well-characterized example of this mechanism, in which defects in the BBSome complex impair leptin receptor trafficking and disrupt melanocortin pathway signaling; the key molecular mechanisms underlying BBS-related obesity are illustrated in Figure 2. In Prader–Willi syndrome, the loss of paternal 15q11–q13 genes (SNORD116, MAGEL2) reduces hypothalamic satiety signaling and impairs prohormone processing, leading to hyperphagia and obesity. In addition, chromatin remodeling defects (e.g., KAT6B, NSD1) alter transcriptional programs controlling neuronal differentiation, appetite circuits, and energy expenditure.

### 1.7. Endocrine Obesity

Obesity due to endocrinopathies occurs in about 2–3% of children, and their identification is essential, as most of these disorders can be treated effectively [98]. Thus, common endocrine disorders such as hypothyroidism, growth hormone deficiency or resistance, and cortisol excess (Cushing’s disease or syndrome) can cause obesity. Polycystic ovarian syndrome (PCOS) may both result from and contribute to obesity. Pseudohypoparathyroidism, due to Gsα inactivating mutations, is also linked to obesity. Albright hereditary osteodystrophy (AHO), an autosomal dominant disorder caused by GNAS1 variants affecting Gsα protein function, presents with short stature, obesity (may be the first presentation), skeletal defects, and impaired olfaction.

### 1.8. Medication-Induced Obesity

Medications such as antipsychotics, antiepileptics, mood stabilizers, anxiolytics, and antidepressants may contribute to weight gain in the pediatric population [99]. Other drug classes that contribute to obesity include endocrine therapies (e.g., insulin, glucocorticoids, and hormonal contraceptives), certain antihypertensives, antihistamines, insomnia treatment, and some chemotherapeutic agents [99].

### 1.9. Acquired Hypothalamic Obesity

The hypothalamus plays a central role in regulating energy balance [100], and hypothalamic obesity (HO) is due to disrupted brain pathways that control appetite, energy use, and hormone signaling. Thus, HO can be caused by brain tumors [101], surgery, or radiation therapy [102], traumatic brain injury [103], and meningitis or ischemic infarction [53]. HO can lead to severe obesity, especially when lesions affect central hypothalamic nuclei tied to melanocortin signaling, leading to increased hunger, lower energy use, and rapid fat accumulation with significant and rapid weight gain, often resistant to conventional weight loss strategies due to leptin resistance, reduced energy expenditure, and autonomic dysfunction [103,104].

### 1.10. Rapid-Onset Obesity with Hypothalamic Dysfunction, Hypoventilation, and Autonomic Dysregulation with Neuroendocrine Tumors (ROHHADNET)

ROHHADNET is a rare syndrome characterized by hyperphagia and rapid and extreme weight gain [105], with no clear genetic determinants to date [106,107,108]. Children with ROHHADNET typically gain weight rapidly, followed over time by hypothalamic dysfunction, hypoventilation, autonomic issues such as impaired bowel motility, and neural crest tumors. Two cases with ROHHAD syndrome treated with high-dose cyclophosphamide showed diminished appetite and stabilized weight [109,110].

## 2. Investigations for Childhood Obesity

### 2.1. General Investigations

Baseline investigations should include measurement of fasting blood glucose, HbA1c, lipids, and liver function tests. Routine laboratory evaluations for endocrine causes of pediatric obesity are typically not recommended unless there is evidence of attenuated growth or rapid weight gain [50,111]. Measuring insulin concentration is not routinely recommended, as it can be highly variable [50,111].

### 2.2. Genetic Testing for Monogenic and Syndromic Obesity

Genetic testing is recommended for patients with obesity-related red flags, which include extreme early-onset obesity (typically below 5 years [50], but in some cases below 2 years [106]), hyperphagia, a family history of extreme obesity, autistic features, developmental delay, syndromic features, or behavioral and cognitive concerns [50,111]. A recent multicenter observational study analyzed height, weight, and BMI from birth to age 5 years in individuals diagnosed with biallelic (likely) pathogenic *LEP*, *LEPR*, *POMC*, *PCSK1*, or *MC4R* variants or monoallelic (likely) pathogenic *MC4R* variants from six European centers [112] and showed that from age 6 months onwards, individuals with biallelic variants can be distinguished from those with monoallelic variants and common obesity, particularly in the presence of syndromic features such as developmental delay, intellectual disability, retinal dystrophy, polydactyly, and hypogonadism. Indeed, a BMI ≥ 24 kg/m^2^ at age 2 years had good diagnostic performance for biallelic variants.

### 2.3. Comorbidities

Childhood obesity is associated with a broad spectrum of metabolic and organ-specific complications, including type 2 diabetes mellitus (T2DM), dyslipidemia, metabolic dysfunction-associated steatotic liver disease (MASLD), and obstructive sleep apnea (OSA), all of which require systematic surveillance (Table 3). Dyslipidemia affects approximately 20–40% of children with obesity. It is typically characterized by elevated plasma free fatty acids, triglycerides, and low-density lipoprotein cholesterol, accompanied by a reduction in high-density lipoprotein cholesterol. MASLD represents the hepatic manifestation of systemic metabolic dysfunction and has emerged as the most common chronic liver disease in children and adolescents with obesity; MASLD is associated with a clustering of adverse metabolic features in children, including insulin resistance, dyslipidemia, impaired glucose regulation, hypertension, and visceral adiposity, which are linked to early vascular and organ dysfunction and confer a substantially increased lifetime risk of type 2 diabetes mellitus and cardiovascular disease [113,114]. T2DM and prediabetes in pediatric populations exhibit a more aggressive disease trajectory than in adults, characterized by profound insulin resistance and accelerated β-cell dysfunction, leading to rapid deterioration of glycemic control and limited long-term therapeutic efficacy of conventional therapies; prediabetes in this age group is similarly highly susceptible to progression, with a high risk of progression to overt diabetes, particularly during puberty when physiological insulin resistance is amplified [115,116,117]. Polycystic ovary syndrome (PCOS) is a common endocrine complication in adolescent females with obesity, frequently presenting with menstrual irregularities and driven predominantly by hyperandrogenism and reduced sex hormone-binding globulin concentrations.

### 2.4. Management of Obesity

Effective management of pediatric obesity begins with identifying the underlying cause (Figure 1) with a patient-centered approach that integrates lifestyle modification, behavioral strategies, pharmacotherapy, and, when appropriate, bariatric surgery. Given the complex, multifactorial etiology of obesity, including genetic susceptibility, behavioral patterns, environmental exposures, and social determinants of health, no single intervention is universally effective [120,121]. The AAP currently emphasizes the importance of initiating intensive lifestyle and behavioral interventions as early as possible, ideally by age six, to promote sustainable habits that prevent the progression of obesity and its associated comorbidities [120,121,122]. These interventions must be adapted to the child’s developmental stage, family-engaged, and culturally sensitive to maximize adherence and equity of access [121].

For adolescents with moderate-to-severe obesity, adjunctive pharmacotherapy using agents such as GLP-1 receptor agonists or phentermine/topiramate has demonstrated additive benefits when combined with lifestyle therapy, though long-term efficacy remains to be established, and potential adverse effects, including gastrointestinal symptoms (e.g., nausea, vomiting), must be carefully considered [121,123]. Bariatric surgery is a safe and effective option, especially for adolescents with severe obesity and obesity-related complications; however, it carries potential risks such as perioperative complications, micronutrient deficiencies, and gastrointestinal symptoms (e.g., dumping syndrome, reflux), and requires lifelong dietary modifications, portion control, and adherence to vitamin and mineral supplementation [120,121,123]. A shared decision-making framework that accounts for individual risk profiles, family readiness, psychosocial context, and patient preferences is central to treatment planning [121,123].

## 3. Management of Polygenic Obesity

### 3.1. Lifestyle Behavioral Interventions

#### 3.1.1. Diet

Dietary interventions remain a cornerstone of pediatric obesity management; however, sustained benefit is most consistently achieved through structured, family-based behavioral approaches rather than specific macronutrient prescriptions [124,125]. Randomized trials demonstrate that interventions emphasizing regular meal scheduling, avoidance of inter-meal grazing, age-appropriate portion control, and slowing of eating pace—particularly when parents act as agents of change—are associated with durable improvements in weight status in children. Slowing eating pace through behavioral “retraining” has shown promise in reducing BMI gain in the short term, although long-term benefits require family engagement and reinforcement [126]. Individualized nutrition counseling incorporating goal setting, behavioral reinforcement, and regular follow-up improves dietary quality and yields modest but clinically meaningful reductions in BMI z-score [127].

In adolescents, flexible dietary strategies such as intermittent energy restriction (IER) [128] and time-restricted eating (TRE) [129] have been proposed to accommodate social and school routines; however, current randomized evidence does not demonstrate superiority over standard dietary counseling, and long-term adherence remains uncertain [128,129,130] when used with continuous glucose monitoring (CGM), showing no significant differences in glycemic variability between the TRE and prolonged eating window groups.

Dietary composition influences metabolic risk, with the substitution of refined carbohydrates by whole grains linked to reduced central adiposity in children [131]. Evidence supporting high-protein or low-glycemic-index diets in adolescents is inconsistent due to poor compliance [132]. Very-low-energy diets (VLEDs) induce rapid short-term weight loss in adolescents but require specialist supervision and are unlikely to be sustainable without follow-up [133]. Consuming sugar-sweetened beverages is associated with higher energy intake and poorer diet quality in children, supporting the need to limit them in pediatric obesity management [134]. Overall, dietary interventions in pediatric obesity need to be developmentally appropriate and integrated into daily life for sustained adherence.

#### 3.1.2. Exercise

Physical activity provides unique cardiometabolic and neurocognitive benefits in pediatric obesity that extend well beyond weight reduction alone [135,136]. In children and adolescents with obesity, engagement in physical activity is frequently limited by reduced cardiorespiratory fitness, impaired motor competence, excess body mass, psychosocial barriers, and obesity-related comorbidities, highlighting the need for an initial conditioning or rehabilitative phase focused on restoring movement tolerance, functional capacity, and confidence before progression to higher training intensities [137,138]. Controlled trials demonstrate that structured exercise programs improve cardiorespiratory fitness and reduce adiposity in youth with obesity, with both moderate-intensity continuous training (MICT) and high-intensity interval training (HIIT) showing efficacy when appropriately supervised [135,139,140,141]. Notably, HIIT has been associated with greater improvements in vascular function and cardiac autonomic regulation compared with MICT in adolescents with obesity, although careful progression and individualization are essential [139,141]. Beyond effects on fitness and body composition, structured exercise has been shown to modulate gut microbiota composition, reduce systemic inflammation, and improve lipid profiles in children with obesity, thereby augmenting the metabolic benefits of dietary interventions [142]. Multicomponent programs integrating aerobic and resistance training are particularly relevant in pediatric populations and have demonstrated modest but clinically meaningful improvements in executive function, academic performance, and mental well-being, especially among adolescents with obesity [136,140,143]. Effective exercise interventions must therefore be developmentally appropriate, age-adapted, and embedded within interdisciplinary and family-based care models to support adherence and long-term behavioral change [137,138].

#### 3.1.3. Behavioral Interventions

The updated United States Preventive Services Task Force (USPSTF) statement in 2024 reinforced behavioral interventions as early as 6 years to establish healthy habits before puberty, including family-based counseling on healthy eating, physical activity, food-labeling literacy goal setting, and self-monitoring of diet and activity behaviors.

Raducha et al. [137] implemented a 12-month interdisciplinary program combining nutritional counseling, physical activity guidance, behavioral therapy, and family involvement. A shorter interval between clinic visits yielded better outcomes in managing pediatric overweight and obesity, but initial gains often slowed or reversed over time, underscoring the importance of maintaining sustained, frequent contact to optimize long-term benefits.

Intensive family-based behavioral treatment (FBT) [124,144], delivered with high fidelity and reinforced through regular follow-up, has consistently demonstrated reductions in BMI. The FABO study integrated behavior change strategies into routine clinical settings and confirmed its feasibility and effectiveness, particularly for treatment-resistant cases [145]. Expanding FBT to include parenting skills training and authoritative limit-setting further enhances adherence and weight outcomes [144]. A variation in this approach, family-based behavioral social facilitation treatment (FBSFT), as evaluated in the FABO study [145], incorporates active problem-solving and social facilitation skills to help families overcome real-world barriers to healthy behaviors. Unlike other family-based interventions, which focus primarily on education and structured follow-up, FBSFT emphasizes navigating social situations, such as managing peer pressure around food, making healthier choices or social events, handing teasing or weight-related stigma, and coordinating consistent routines across school, home, and extracurricular settings, setting practical home rules, and addressing daily challenges, making it particularly valuable for treatment-resistant cases. The Bright Bodies program [146], an intensive, family-based behavioral lifestyle intervention combining nutrition education, physical activity, and behavior modification, has demonstrated cost-effective and sustained reduction in BMI.

Participatory approaches, such as structured family meal prescriptions and grocery-based incentives [147], have improved the home food environment and reduced BMI z-scores in the short term [147,148]. The “Healthy Lifestyles for Children and Adolescents” program [149] in China significantly reduced overweight and obesity rates through integrated health education, physical activity, and behavioral skills [149]. In contrast, whilst the Texas Sprouts program [150] increased vegetable intake, it did not impact BMI [150], and the Kids Obesity Prevention (KOP) program [151] improved health literacy and self-regulation, but did not affect weight [151].

Digital health tools [151,152,153] augment behavioral interventions, and hybrid online FBT [152], mobile apps [153], and gamified platforms [151] have shown promise in enhancing dietary habits, screen time management, and parental self-efficacy [152,153]. The MINISTOP 2.0 app [154] improved diet quality and reduced screen time in preschoolers, but had no impact on the BMI [154]. Active video game (AVG) interventions have also shown promise [155,156]. A five-month AVG plus multicomponent training program [155] improved muscular fitness, motor competence, and physical activity in children with overweight or obesity [155]. The W8Loss2Go app [156], which applies an addiction-based behavioral model to curb snacking and manage meal size, achieved behavioral improvements but no significant BMI reduction compared to in-person care [156]. Mack et al. [151] integrated digital games into classroom lessons and achieved a remarkably low drop-out rate of 3.6% with a significant improvement in lean mass and sedentary time. The gamified approach likely sustained motivation and engagement, two major challenges in pediatric obesity management.

### 3.2. Pharmacotherapy

**Figure 3:**
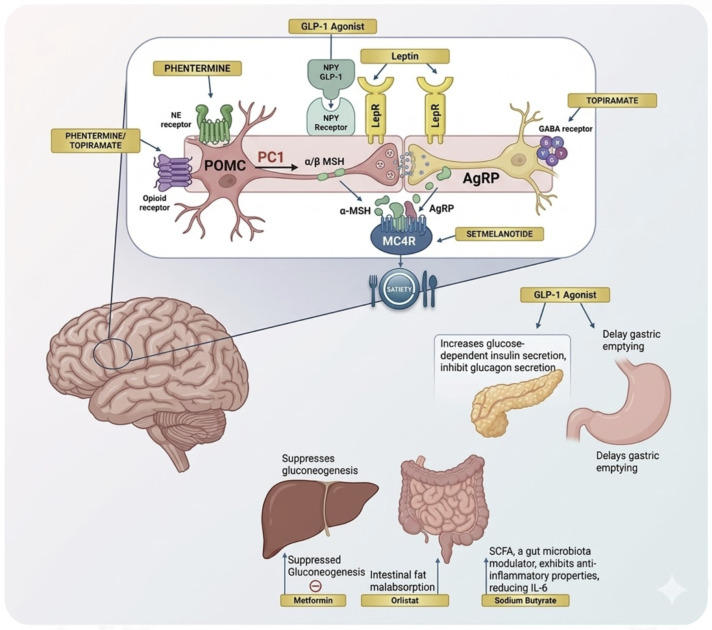
Anti-obesity medication in pediatric patients and their mechanisms of action. Phenteramine increases norepinephrine (NE) in the central nervous system (CNS), suppressing appetite through hypothalamic stimulation. The combination of phentermine/topiramate enhances norepinephrine release, augments gamma-aminobutyric acid (GABA) activity, and inhibits α-amino-3-hydroxy-5-methyl-4-isoxazolepropionic acid (AMPA)/kinase excitatory glutamate receptors, reducing appetite. Orlistat is a gastrointestinal (GI) lipase inhibitor that blocks the digestion and absorption of up to 30% of dietary fat. Sodium butyrate is a short-chain fatty acid (SCFA) that modulates gut microbiota, exerts anti-inflammatory effects, and reduces interleukin-6 (IL-6) levels. Exenatide XR is a glucagon-like peptide-1 receptor agonist (GLP-1 RA) that acts on the hypothalamus and reward centers of the brain to increase glucose-dependent insulin secretion, inhibit glucagon secretion, and delay gastric emptying. Liraglutide is another GLP-1 RA with a similar mechanism to exenatide, approved in specific doses for type 2 diabetes mellitus (T2DM) and obesity in children aged six years and above. Dulaglutide is a weak GLP-1 RA that increases satiety, enhances glucose-dependent insulin secretion, and delays gastric emptying. Semaglutide is a potent GLP-1 RA with prolonged action, improving satiety, reducing appetite, and regulating glucose metabolism. Tirzepatide is a dual glucose-dependent insulinotropic polypeptide (GIP) and GLP-1 receptor agonist under investigation, which suppresses appetite and increases insulin secretion. Metformin is a biguanide that reduces hepatic gluconeogenesis, improves peripheral insulin sensitivity, and promotes modest weight reduction. CNS: central nervous system; NE: norepinephrine; GABA: gamma-aminobutyric acid; AMPA: α-amino-3-hydroxy-5-methyl-4-isoxazolepropionic acid; GI: gastrointestinal; SCFA: short-chain fatty acid; IL-6: interleukin-6; GLP-1 RA: glucagon-like peptide-1 receptor agonist; T2DM: type 2 diabetes mellitus; GIP: glucose-dependent insulinotropic polypeptide.

Anti-obesity medications should always be administered in conjunction with lifestyle modification [157]. The updated United States Preventive Services Task Force (USPSTF) statement in 2024 reinforced intervention as early as 6 years to establish healthy habits before puberty [122]. Although several medications have been approved by the FDA, including Orlistat (2003), Liraglutide (2020), Semaglutide (2022), and phentermine/topiramate combination (2022) [122], long-term benefits of these therapies are not established, and weight either plateaus or rebounds after medication discontinuation [122]. In addition, these pharmacotherapies may be associated with adverse effects such as GIT symptoms (e.g., nausea, vomiting, and diarrhea) and may require long-term adherence to maintain benefits, with weight regain commonly observed after discontinuation. In 2023, the American Academy of Pediatrics (AAP) [50] recommended that pediatricians should prescribe weight loss medications to adolescents 12 years and older with BMI ≥ 95th percentile, together with health behavior and lifestyle modification [50]. The approved anti-obesity medications and their mechanism of action are shown in Table 4 and Figure 3. In addition, GLP-1 receptor agonists have emerged as key pharmacologic options in adolescent obesity. The seminal, randomized, placebo-controlled trial by Kelly et al. [158] demonstrated that Liraglutide (3.0 mg daily) produced significantly greater reductions in BMI and higher proportions achieving clinically meaningful weight loss thresholds of ≥5% and ≥10% of baseline body weight compared to placebo in adolescents aged 12–17 years. Building on this, the STEP TEENS trial by Weghuber et al. [159] showed that once-weekly Semaglutide (2.4 mg) resulted in a mean BMI reduction of ~16% over 68 weeks, with substantially greater weight loss and metabolic improvements than placebo in adolescents with obesity. However, these trials also reported common adverse effects, particularly GIT symptoms, and the durability of benefits beyond the treatment period remains uncertain, highlighting the potential for weight regain after therapy cessation. These two key trials approved the evidence base supporting FDA approvals of Liraglutide and Semaglutide for youth ≥ 12 years old and present major advances in pharmacotherapy for pediatric obesity.

### 3.3. Bariatric Surgery

**Figure 4:**
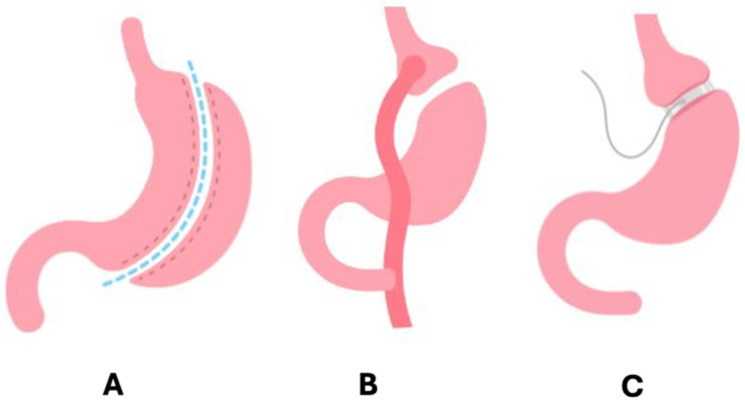
Major types of bariatric surgical procedures: (**A**) LSG (laparoscopic sleeve gastrectomy), where a portion of the stomach is removed, leaving a smaller sleeve-shaped stomach to restrict food intake; (**B**) RYGB (Roux-en-Y gastric bypass), where the stomach is divided into a small upper pouch and a larger lower remnant, with the small intestine connecting to the pouch bypassing the majority of the stomach and upper small intestine to limit food absorption and intake; and (**C**) AGB (adjustable gastric band), where an inflatable band around the upper portion of the stomach creates a smaller pouch to restrict food intake.

There has been a 5-fold increase in the number of bariatric surgery procedures in adolescents from 1997 to 2003 [169,170]. There are three main types of bariatric procedures: laparoscopic sleeve gastrectomy (LSG) (Figure 4A), Roux-en-Y gastric bypass (RYGB) (Figure 4B), and adjustable gastric band (AGB) (Figure 4C).

The Teen Longitudinal Assessment of Bariatric Surgery Study (TEEN-LABS) is the largest study to date to investigate the long-term safety and efficacy of bariatric surgery in adolescents (≤19 years of age) [171]. Sleeve gastrectomy is the most common option for adolescents due to the lower risk of micronutrient deficiencies. The RYGB procedure reduces the capacity of the stomach, causing restriction of caloric intake and resulting in malabsorption of food, vitamins, and minerals. The AGB is not an FDA-approved procedure for adolescents younger than 18 years [172].

The American Society for Metabolic and Bariatric Surgery (ASMBS) has recommended bariatric surgery in adolescents with a BMI ≥ 35 kg/m^2^ with moderate-to-severe OSA (apnea-hypopnea index > 15), T2DM, pseudotumor cerebri, or severe and progressive steatohepatitis, or a BMI ≥ 40 kg/m^2^ [173,174]. Candidates should have reached physical maturity (Tanner stage IV), have a history of efforts to lose weight through diet and physical activity, demonstrate willingness to follow pre- and postoperative management plans, and have family support with an understanding of the risks and benefits of surgery. On the other hand, exclusion criteria for bariatric surgery in adolescents [173,175] includes a medically treatable cause for obesity; substance abuse; medical, psychiatric, psychological, or cognitive conditions; current or planned pregnancy within 12–18 months of the procedure; inability of the patient or families to understand the risks and benefits of the procedure; financial constraints; inability to attend required follow-up visits; or inability to adhere to lifelong nutritional supplementation.

Long-term follow-up from the TEEN-LABS consortium demonstrated durable and substantial weight loss at 5 years following bariatric surgery, accompanied by high rates of remission of obesity-related comorbidities, including T2DM and hypertension. Importantly, metabolic improvements were sustained despite some weight regain, although the need for ongoing monitoring for micronutrient deficiencies and additional abdominal procedures was noted. These findings underscore both the effectiveness and the necessity for long-term surveillance after adolescent bariatric surgery [176].

In a randomized controlled trial of adolescents aged 15.9 ± 1.0 years with severe obesity (weight 129 ± 18.5 kg) who underwent adjustable gastric banding and lifestyle intervention, weight was reduced by −8.83 ± 5.18 at 6 months and −11.23 ± 7.76 at 12 months with a significant improvement in HOMA-IR index at 6 (+3.1 (2.4–4.6)) and 12 (+4.2 (2.9–5.4)) months [177]. Cardiovascular risk was significantly lower one year after bariatric surgery (Teen-LABS trial) and was sustained at 5-year follow-up (TODAY trial), when compared to medical therapy [178].

## 4. Treatment of Monogenic Obesity

### 4.1. Non-Pharmacological Treatment Options

Patients with monogenic obesity have defects in the hunger–satiety feedback mechanisms, which will limit the success of any lifestyle interventions that restrict calorie intake due to the severe hyperphagia in these patients. In one study, children with MC4R pathogenic variants initially lost weight in a lifestyle intervention program (exercise, behavior/nutritional counseling) but had difficulty maintaining their weight loss [179]. In another study, carriers of MC4R pathogenic variants failed to reduce their BMI with lifestyle intervention [180]. Patients with congenital leptin deficiency have not responded to weight loss programs and psychotherapy [181].

### 4.2. Metreleptin for Congenital Leptin Deficiency

Metreleptin is a recombinant form of leptin that is FDA-approved for the treatment of patients with congenital leptin deficiency. Subcutaneous administration of Metreleptin leads to a reduction in food intake due to a profound effect on the patient’s pre-occupation with food [182], resulting in weight loss with a selective loss of adipose tissue [183] and improvement in hyperinsulinemia, hyperlipidemia, and liver steatosis [184].

The recent classification of leptin gene variants into classical hormone deficiency, biologically inactive hormone, and antagonistic hormone has further aided in fine-tuning the treatment of patients with congenital leptin deficiency [185]. Patients with the classical hormone deficiency and biologically inactive hormone are typically treated with a starting dose of 0.03 mg/kg of lean body mass per day, whilst those with the antagonistic variant leptin need to be started on a higher dose of Metreleptin [186]. Whilst Metreleptin is the ideal treatment for patients with congenital leptin deficiency, most patients do not have access to this treatment due to cost and availability. A study from Pakistan [187] showed that children with congenital leptin deficiency who could not be treated with Metreleptin had a high mortality and morbidity due to pulmonary and gastrointestinal infections. Recently, a fully human monoclonal antibody, REGN4461 (mibavademab), that activates the human leptin receptor has been shown to normalize body weight, food intake, blood glucose, and insulin sensitivity in obese leptin knockout mice [188]. In an adult patient with atypical partial lipodystrophy, the administration of REGN4461 led to notable improvements in circulating triglycerides and hepatic steatosis [188].

### 4.3. Setmelanotide Therapy for Monogenic Obesity Due to Leptin Receptor (LEPR), Proopiomelanocortin (POMC), and Proprotein Convertase Subtilisin/Kexin Type 1 (PCSK1)

Pathogenic variants in the genes encoding for LEPR, POMC, and PCSK1 affect the MC4R pathway and are associated with hyperphagia, leading to early-onset obesity. Multiple endocrinopathies, including adrenocorticotropic hormone deficiency, hypothyroidism, hypogonadism, hypopigmentation, hypoglycemia, and gastrointestinal involvement, can also occur in patients with PCSK1 variants. Setmelanotide crosses the blood–brain barrier and acts on the hypothalamus [189], mimicking endogenous alpha-MSH (anorexigenic action), and restores normal signaling via the MC4R to regulate satiety and appetite. Setmelanotide is currently FDA-approved for treating children over 6 years of age with obesity due to genetic mutations in LEPR, POMC, PCSK1, and BBS [190].

In an investigator-initiated open-label study, Setmelanotide was administered to two adult patients with POMC deficiency who developed obesity at the age of 4 years [66]. The first patient lost 51kg after 42 weeks, and the second patient lost 20.5 kg over 12 weeks, with both patients reporting a significant reduction in hunger scores. In another study of three patients (ages 23, 22, and 14 years) with severe obesity due to LEPR gene mutations administered Setmelanotide, there was a significant reduction in hyperphagia and weight over 45–61 weeks [191].

Two pivotal open-label multicenter phase 3 trials in patients aged 6 years or older with POMC (10 patients) or LEPR (11 patients) deficiency [65] showed that 80% of participants in the POMC trial and 45% of participants in the LEPR trial achieved at least 10% weight loss at approximately 1 year, with an improvement in the quality of life as early as 5 weeks after starting treatment [192]. The recommended starting dose of Setmelanotide for children aged between 6 and <12 years is 1 mg (0.1 mL) injected subcutaneously daily for two weeks, which, if tolerated, can be titrated to 2 mg. The starting dose in patients 12 years of age and older is 2 mg, which, if tolerated, can be titrated to 3 mg.

More recently, a phase 3, open-label, multicenter trial enrolled eligible patients aged 2–5 years (mean age 3.6 years) who had hyperphagia and obesity due to biallelic POMC (including PCSK1) or LEPR variants or genetically confirmed BBS [193]. Subcutaneous Setmelanotide was administered once daily for 52 weeks, starting at 0·5 mg with doses increasing every 2 weeks in 0·5 mg increments, and achieved a significant reduction in hunger and BMI Z scores in all patients.

Hypersensitivity with blistering, burning, hives, itching, and discoloration of the skin at the injection site can occur with Setmelanotide, with the most common specific side effect being skin hyperpigmentation (skin tanning and hair darkening) due to the activation of melanocortin receptors on melanocytes, leading to an accumulation of melanin, which is reversible when the drug is stopped [194].

### 4.4. MC4R-Related Obesity and Treatment Options

The GLP-1 (Glucagon-Like Peptide 1) agonist Liraglutide showed a significant reduction in weight and improvement in fasting and postprandial glucose in 14 adults with obesity with MC4R pathogenic variants [59], and this has been confirmed in several case reports, including pediatric patients [195,196,197]. GLP-1 agonists suppress appetite via an MC4R-independent pathway/s and do not require functional MC4R receptors. Another potential treatment for patients with MC4R obesity is Setmelanotide [198], which may seem illogical as Setmelanotide requires functional MC4R for its action. However, Setmelanotide has been shown to restore surface expression of MC4R on cell lines of some MC4R pathogenic variants [198]. In a phase 1B trial in adults with obesity due to MC4R pathogenic variants, Setmelanotide led to weight loss over 28 days [198].

### 4.5. Bariatric Surgery for Monogenic and Syndromic Obesity Disorders

The role of bariatric surgery in patients with monogenic and syndromic obesity is uncertain. Laparoscopic sleeve gastrectomy has been reported to be safe and effective for weight loss in the short-term in a large cohort of children below the age of 14 years with all forms of obesity [199,200], including monogenic obesity. A 2-year follow-up study [201] of children with monogenic obesity due to mutations in MC4R, POMC, and PCSK1 showed that the total body weight loss of approximately 25–30% after RYGB surgery did not differ significantly when compared with patients without a molecular diagnosis. Patients with MC4R mutations had better weight loss after primary Roux-en-Y gastric bypass compared to sleeve gastrectomy. A retrospective analysis [202] over 19 years in eight patients with pathogenic variants in the LEPR, POMC, and MC4R who had undergone bariatric surgery (including RYGB) showed weight loss initially, but then substantial weight regain on follow-up, possibly related to factors such as dilatation of the gastric pouch, loosening of restrictive components, or maladaptive eating behaviors. There is limited evidence for bariatric surgery as a treatment option for children with monogenic obesity [203].

### 4.6. Treatment of Syndromic Forms of Obesity

Many different syndromes lead to obesity, including PWS, BBS, and Alstrom syndrome. Thus, in PWS, food-seeking behavior and hyperphagia both lead to early-onset obesity. Clinical trials using oxytocin, an unacylated ghrelin analog, diazoxide choline controlled-release tablet, Setmelanotide, and GLP-1 receptor agonists have all reported varying degrees of success (Table 2) [204].

We recently reported improvement in food-related behaviors with significant weight loss (11.5% over 18 weeks) and complete resolution of type 2 diabetes with Semaglutide and Methylphenidate combination therapy in a child with PWS [87]. In a multicenter double-blind placebo-controlled phase 3 trial in patients aged 6 years or older with BBS, treatment with Setmelanotide 3 mg daily led to a 10% bodyweight reduction over 52 weeks, but there was no effect in patients with Alstrom syndrome [89]. A recent expert review of clinical trials in children > 6 years of age with BBS concluded that Setmelanotide can dramatically improve hyperphagia, reduce body mass, and improve comorbidities [205], along with multiple health-related quality of life measures [206], including emotional and physical well-being [207]. Setmelanotide may also decrease the severity of metabolic syndrome and risk of future obesity-related comorbidities in those with BBS [208]. In a recent study [209], Setmelanotide in 12 patients with Smith–Magenis syndrome aged between 11 and 39 years showed no significant effect on weight, indicating that obesity in these patients does not involve dysfunction of the MC4R pathway.

### 4.7. Treatment for Hypothalamic Obesity

The treatment of hypothalamic obesity has shown limited success [101] with dextro-amphetamine and somatostatin analogs, reporting moderate weight loss [210,211,212]. The GLP-1 receptor agonist, Semaglutide, in four patients with hypothalamic obesity due to craniopharyngiomas showed an improvement in eating behavior and a reduction in weight [213]. More recently, in a phase 2, open-label, multicenter trial in patients aged 6 to 40 years with obesity and a history of hypothalamic injury or diagnosis of a non-malignant tumor affecting the hypothalamus treated with surgery, chemotherapy, or radiation, daily subcutaneous Setmelanotide (3.0 mg) led to a reduction in hunger scores and 15% reduction in BMI over 16 weeks [214].

## 5. Conclusions

Childhood obesity is a complex and heterogeneous condition arising from an interplay of nutritional, environmental, behavioral, genetic, and metabolic factors. Its classification into polygenic, monogenic, syndromic, and hypothalamic forms underscores the need for a tailored approach to diagnosis and treatment. Whilst lifestyle modification remains the cornerstone of management, it has shown limited efficacy. Growing evidence supports genetic evaluation and personalized pharmacological therapies. As new therapeutic options emerge, ensuring equitable access and sustained implementation in real-world settings will be key to mitigating the impact of this escalating global health challenge.

## Figures and Tables

**Table 1:** Underlying genes, inheritance patterns, primary phenotypic characteristics, and treatment options in monogenic obesity.

#	Gene Symbol	Gene Name	OMIM #	Mode of Inheritance	Phenotype	Treatment	References
1	*MC4R*	Melanocortin 4 receptor	155541	AD, AR	Severe EOO Hyperphagia Linear growth Hypotension	Liraglutide (adults) Setmelanotide	[59,60]
2	*LEP*	Leptin	164160	AR	Severe EOO Hyperphagia Hypogonadism Immune dysfunction Insulin resistance Dyslipidemia Behavioral and cognitive issues	Metreleptin	[60,61]
3	*LEPR*	Leptin receptor	601007	AR	Severe EOO Hyperphagia Hypogonadism Infertility Insulin resistance Dyslipidemia Behavioral and cognitive impairments Immune dysfunction Autonomic dysfunction	Setmelanotide	[61,62,63]
4	*POMC*	Proopiomelanocortin	176830	AD, AR, UK	Severe EOO Hyperphagia Adrenal dysfunction Hypopigmentation	Setmelanotide	[63,64,65,66]
5	*PCSK1*	Proprotein convertase subtilisin kexin type 1	162150	AR, UK	EOO Diarrhea Hypoglycemia Insulin resistance Developmental Delay Immune system dysfunction	Setmelanotide	[62,65]
6	*GNAS*	Guanine nucleotide binding protein alpha stimulating	139320	AD	Obesity Short stature Osteodystrophy Brachydactyly Pseudohypoparathyroidism	No treatment is currently available	[63]
7	*MRAP2*	Melanocortin 2 receptor accessory protein 2	615410	AD	EOO Adrenal insufficiency Hypogonadism	No treatment is currently available	[67]
8	*SH2B1*	Src homology 2B1	608937	AD	Severe EOO Hyperphagia Cognitive impairments Neurological disorders	No treatment is currently available	[68]
9	*SIM1*	Single-minded homolog 1	603128	AD	Severe EOO Hyperphagia Developmental and behavioral issues Autonomic dysfunction Hypothalamic dysfunction	No treatment is currently available	[69]
10	*ADCY3*	Adenylate cyclase 3	600291	AR	EOO Common Obesity T2D Hypogonadotropic Hypogonadism Olfactory dysfunction Behavioral disorders	No treatment is currently available	[70]
11	*BDNF*	Brain-derived neurotrophic factor	113505	AD	EOO Mood disorders Neurological disorders	No treatment is currently available	[71]
12	*NTRK2*	Neurotrophic receptor tyrosine kinase 2	600456	AD	Obesity Neurodevelopmental disorders Behavioral and psychiatric features Endocrine abnormalities Intellectual disability	No treatment is currently available	[72]
13	*DYRK1B*	Dual-specificity tyrosine phosphorylation-regulated kinase 1B	604556	AD	Abdominal Obesity Insulin resistance T2D Hypertension Neurodevelopmental disorders	No treatment is currently available	[73]
14	*PHIP*	Pleckstrin homology domain-interacting protein	612870	AD	Obesity, developmental delay, hypotonia	No treatment is currently available	[74]
15	CPE	Carboxypeptidase E	619326	AR	Obesity, diabetes, hypogonadism, and intellectual disability	No treatment is currently available	[62]
16	NCOA1	Nuclear receptor coactivator 1	602691	AD	Obesity, Hypogonadism/pubertal delay	No treatment is currently available	[75]
17	SEMA3E	Semaphorin-3A	608166	AD	Obesity, anosmia, and developmental delay	No treatment is currently available	[76]

EOO: Early-onset obesity, AD: Autosomal dominant, AR: Autosomal recessive, T2D: Type 2 diabetes, and UK: Unknown.

**Table 2:** Underlying genes, incidence, primary phenotypic characteristics, and treatment options in syndromic forms of obesity.

Name of Syndrome	Causal Gene/Region	Incidence	Phenotype	Treatment	References
Prader–Willi syndrome	15q11. 2-q13 (deletion/methylation)	1 in 15,000 to 1 in 20,000	Severe obesity hyperphagia Hypotonia Developmental delay Intellectual disabilities Hypogonadism Diabetes mellitus	Beloranib Oxytocin Setmelanotide Diazoxide choline-controlled release Livoletide Exenatide Combination of Methylphenidate and Semaglutide	[78,79,80,81,82,83,84,85,86,87]
Bardet–Biedl syndrome	BBS1-BBS22	1 in 100,000 to 1 in 160,000	Obesity, retinal degeneration, and polydactyly Intellectual disabilities Kidney abnormalities Genital abnormalities Diabetes mellitus Cardiovascular abnormalities Facial dysmorphism	Setmelanotide	[88,89]
Alstrom syndrome	ALMS1	1 in 1,000,000 to 1 in 2,000,000	Obesity Type 2 diabetes mellitus Hearing loss Vision problems Cardiomyopathy	No treatment is currently available	[90]
Albright hereditary osteodystrophy	GNAS	1 in 100,000 to 1 in 200,000	Obesity Pseudohypoparathyroidism Short stature Brachydactyly Subcutaneous ossification Hypothyroidism Hypogonadism Intellectual disability	No treatment is currently available	[91]
Schaaf–Yang syndrome	MAGEL2	N/A	Obesity Hyperphagia Developmental delay Facial dysmorphism Intellectual disability Autism Hypogonadism Hypotonia	No treatment is currently available	[92]
Smith–Magenis syndrome	RAI1	1 in 25,000 to 1 in 50,000	Obesity Hyperphagia Intellectual disabilities Speech delay	No treatment is currently available	[93]
Cohen syndrome	VPS13B	1 in 100,000 to 1 in 200,000	Obesity, Developmental delay Vision impairment Neutropenia	No treatment is currently available	[94]
Fragile-X syndrome	FMR1	1 in 4000 to 1 in 6000 live births in males, and about 1 in 8000 to 1 in 10,000	Obesity Intellectual disability Speech and language delay Autism behavior	No treatment is currently available	[95]
Down syndrome	NA	~1 in 700 live births (varies with maternal age)	Obesity, intellectual disability, early Alzheimer’s risk, congenital heart defects, gastrointestinal atresias, hypotonia, hearing/vision issues, and endocrine disorders	No treatment is currently available	[96]
Turner syndrome	NA	~1 in 2500 live female births	Obesity, ovarian dysgenesis, short stature, webbed neck, broad chest, hearing loss, autoimmune thyroiditis, and risk of aortic dissection	No treatment is currently available	[97]

BBS1-BBS22: Bardet–Biedl syndrome; ALMS1: Alstrom syndrome 1; GNAS: Guanine nucleotide-binding protein, Aloha-stimulating activity polypeptide; MAGEL2: Melanoma Antigen Family-Like 2; RAI1: Retinoic Acid Induced 1; VPS13B: Vacuolar Protein Sorting 13 Homolog B; FMR1: Fragile X Messenger Ribonucleoprotein 1.

**Table 3:** Screening for comorbidities in children with overweight and obesity.

Guidelines		Dyslipidemia with		Glucose Metabolism with		MASLD with		HTN with	
		Overweight *	Obesity *	Overweight	Obesity	Overweight	Obesity	Overweight	Obesity
AAP 2023 [50]	Recommendation	Age ≥ 10 years: All	Age 2–9: if positive risk factors ≥10 years: All	Not recommended unless positive risk factors in children aged ≥ 10 years	Age 2–9 years: Not recommended unless positive risk factors for those aged ≥ 10 years: All	Not recommended unless positive risk factor in children aged ≥ 10 years	Age 2–9 years: Not recommended unless positive risk factor for those aged ≥ 10 years: All	Start at 3 years of age	
			Positive risk factors -Family history of obesity-related diseases -Elevated BP -Tobacco use	Positive Risk Factors -Family history -History of GDM -Signs of insulin resistance (such as acanthosis nigricans) -Use of obesogenic psychotropic medications		Positive Risk Factors -Family history of MASLD, central adiposity, signs of insulin resistance, prediabetes or diabetes mellitus, dyslipidemia, and sleep apnea			
	Test	Fasting lipid profile		FPG, OGTT, HbA_1c_ Against Routine insulin measurement		ALT	ALT and US	BP	
	Repetition	In 2 years: if normal In 6 to 12 months: if abnormal		In 2 years: Normal In 12 months: HbA1c 5.7 to < 6.0% +/− risk factors 3–6 months: HbA1c ≥ 6.0 to 6.4%		In 2 years: if normal In 3 to 6 months: if abnormal at twice the upper limit of normal or greater (ALT ≥ 52 IU/L for boys and ALT ≥ 44 IU/L for girls (NASPGHAN)		Every visit	
NICE 2022 [118]	Recommendation	Recommended for both Overweight and Obese No age preference		Recommended for both Overweight and Obese No age preference		Recommended for both Overweight and Obese No age preference		Start at 3 years of age	
	Test	Fasting lipid profile		FPG OGTT Fasting insulin		LFT		BP	
ES 2019 [119]	Recommendation	Recommended for both Overweight and Obese No age preference		Recommended for both Overweight and Obese No age preference		Recommended for both Overweight and Obese No age preference		Start at 3 years of age	
	Test	Fasting lipid profile		FPG, OGTT, HbA_1c_ over routine insulin measurement		ALT		Start at 3 years of age	

* Based on the AAP 2023 guidelines, weight status is defined using BMI-for-age percentiles based on sex-specific growth charts. For 2–19 years. Overweight is defined as a BMI between the 85th and <95th percentile, whereas obesity is a BMI ≥ 95th percentile for age and sex. AAP: American academy of pediatrics; NICE: National institute for health and care excellence; ES; BP: blood pressure; GDM: gestational diabetes; FPG: fasting plasma glucose; OGTT: oral glucose tolerance test; HbA1c: glycated hemoglobin; MASLD: metabolic dysfunction-associated steatotic liver disease; ALT: alanine transferase; US: ultrasound; NASPGHAN: North American Society For Pediatric Gastroenterology, Hepatology and Nutrition; LFT: liver function test; HTN: hypertension; TC: total cholesterol; LDL: low-density lipoprotein; HDL: high-density lipoprotein; TG: triglycerides; T2DM: type 2 diabetes mellitus; HbA1c: glycated hemoglobin. Normal range for TC: <170 mg/dL (<4.4 mmol/L), LDL: <110 mg/dL (<2.8 mmol/L), HDL: >45 mg/dL (>1.2 mmol/L), TG: 0–9 years: <75 mg/dL (<0.85 mmol/L) and 10–19 years: <90 mg/dL (<1.0 mmol/L). Borderline high for TC: 170–199 mg/dL (4.4–5.1), LDL: 110–129 mg/dL (2.8–3.3 mmol/L), TG: 0–9 years: 75–99 mg/dL (0.85–1.0 mmo/L) and 10–19 years: 90–129 mg/dL (1.0–1.4 mmol/L). High for TC: ≥ 200 mg/dL (≥5.2 mmol/L), LDL: ≥130 mg/dL (≥3.4 mmol/L), TG: 0–9 years: ≥100 mg/dL (≥1.1 mmol/L) and 10–19 years: ≥130 mg/dL (≥1.5 mmol/L). Normal range for FPG: <100 mg/dL, prediabetes/impaired glucose tolerance: 100–126 mg/dL, T2DM: >126 mg/dL, normal range for 2 h OGTT: <140 mg/dL, prediabetes/impaired glucose tolerance: 140–200 mg/dL, T2DM: >200 mg/dL, normal range for HbA1c: <5.7%, prediabetes/impaired glucose tolerance: 5.7–6.5%, T2DM: ≥6.5 mg/dL, normal range for ALT for boys: ≥52 IU/L and girls ≥44 IU/L. Normal range for HTN: 3–13 years: 90th percentile, elevated: 90–95th percentile, stage 1: 95th +12 mmHg percentile, and stage 2: >95th percentile +12 mmHg. Normal range for HTN: ≥13 years: 120/80 mmHg, elevated: 120/80—130/80 mmHg, stage 1: 130/80–140/90 mmHg, and stage 2: ≥140/90 mmHg.

**Table 4:** Pharmacologic therapies for childhood and adolescent obesity.

Acting	Medication	Class/Mechanism	Dose/Route	Eligible Age Group	Adverse Effects
CNS	Phentermine in combination with LSM [160]	Increases norepinephrine	15 mg, OD, PO	>16	Xerostomia, insomnia, headache, constipation
	Phentermine/Topiramate [157,160]	Increases norepinephrine, augments GABA, and inhibits AMPA/kinase excitatory glutamate receptors	Low dose: 7.5 mg/46 mg, OD, PO high dose: 15 mg/92 mg, OD, PO	12	Paresthesia, xerostomia, constipation, and dysgeusia
GIT	Orlistat [157]	A GI lipase inhibitor blocks the digestion and absorption of up to 30% of dietary fat	120 mg, TID, PO	12	Fecal spotting, fecal urgency, and steatorrhea
	Sodium Butyrate [161]	SCFA, gut microbiota modulator, produces anti-inflammatory properties and reduces IL-6, and may contribute to body-weight regulation by improving gut barrier integrity, enhancing insulin sensitivity, promoting satiety signaling through gut–brain pathways.	20 mg/kg/day, PO	5–17	Mild nausea, headache
Hormone-based	Exenatide XR * [162,163,164]	GLP-1 RA acts in the hypothalamus and reward centers of the brain to increase glucose-dependent insulin secretion, inhibit glucagon secretion, and delay gastric emptying	2 mg, once weekly, SC	10	
	Liraglutide * (* 1.2 and 1.8 mg doses FDA approved for T2DM) [157,158,164,165]		3 mg, OD, SC	6	Nausea, vomiting, constipation, and abdominal pain
	Dulaglutide * [164,166]		1.5 mg, once weekly SC	10	
	Semaglutide [157,164,167]		2.4 mg, once weekly, SC	12	
	Tirzepatide ^ [168]	Dual GIP/GLP-1RA to suppress appetite and increase insulin secretion	Under investigation (NCT05260021) in combination with metformin or basal insulin, or both	10	
Others	Metformin * [157]	Biguanide, an insulin sensitizer	Up to 1 g, BID (if tolerated), PO	10	Nausea, vomiting, constipation

CNS: central nervous system, GIT: gastrointestinal tract, LSM: lifestyle modification, T2DM: type 2 diabetes mellitus, GI: gastrointestinal, SCFA: short chain fatty acid, IL-6: interleukin-6, GIP: gastric inhibitory polypeptide, GLP-1 RA: glucagon like peptide 1 receptor agonist, TID: 3 times daily, OD: once daily, BID: twice daily, PO: by mouth, and SC: subcutaneous. * FDA-approved only for T2DM. ^ Under investigation in children with obesity and T2DM, but not yet FDA-approved.

## Data Availability

No new data were created or analyzed in this study. Data sharing is not applicable to this article.
