# Correction: Gad et al. Management of Childhood Obesity. *Int. J. Mol. Sci.* 2026, *27*, 3528

**DOI:** 10.3390/ijms27125240

**Published:** 2026-06-10

**Authors:** Hoda Gad, Hajar Dauleh, Idris Mohammed, Rayaz A. Malik, Khalid Hussain

**Affiliations:** 1 Lunenfeld-Tanenbaum Research Institute, Mount Sinai Hospital, Toronto, ON M5T 3H7, Canada; 2 Endocrinology Department, Sidra Medicine, Doha P.O. Box 26999, Qatar; 3 Department of Medicine, Weill Cornell Medicine-Qatar, Doha P.O. Box 24144, Qatar; 4 Institute of Cardiovascular Medicine, University of Manchester, Manchester M13 9PL, UK

## Error in Figure

In the original publication [1], there was a mistake in Figure 1 as published. The titles for Alström syndrome and Bardet–Biedl syndrome in Figure 1, titled “**Figure 1.** Diagnostic approach and phenotypic spectrum of pediatric obesity.” were inadvertently altered.

The corrected Figure 1 appears below.

The authors apologize for any inconvenience caused and state that the scientific conclusions are unaffected. This correction was approved by the Academic Editor. The original publication has also been updated.

## Figures and Tables

**Figure 1:**
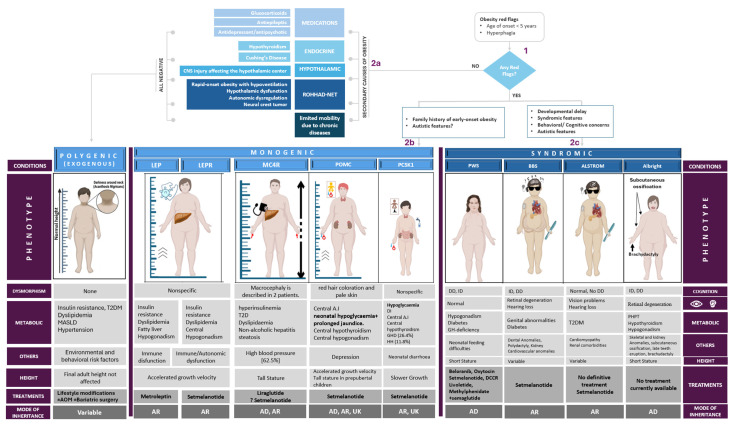
Diagnostic approach and phenotypic spectrum of pediatric obesity. Evaluation begins with screening for red flags (age of onset < 5 years and hyperphagia), which should prompt consideration of pathogenic causes other than polygenic obesity, including monogenic and syndromic forms. Secondary causes of obesity—such as medication-induced, endocrine, and hypothalamic disorders, as well as conditions associated with limited mobility—should also be excluded. The diagnostic pathway is organized into sequential steps: (1) initial evaluation for red flags, followed by (2a) assessment for secondary causes of obesity, (2b) evaluation for monogenic obesity, and (2c) evaluation for syndromic obesity. The lower panel summarizes key clinical characteristics, metabolic complications, and treatment approaches across the major categories of pediatric obesity. Polygenic obesity represents the most common form and is associated with environmental and behavioral risk factors. Monogenic obesity includes defects in the leptin–melanocortin pathway (LEP, LEPR, MC4R, POMC, and PCSK1), while syndromic obesity occurs in genetic disorders such as Prader–Willi syndrome (PWS), Alström syndrome, Bardet–Biedl syndrome (BBS), and Albright hereditary osteodystrophy. Abbreviations: LEP, leptin gene deficiency; LEPR, leptin receptor gene deficiency; MC4R, melanocortin 4 receptor deficiency; POMC, proopiomelanocortin deficiency; PCSK1, proprotein convertase subtilisin/kexin type 1 deficiency; PWS, Prader–Willi syndrome; BBS, Bardet–Biedl syndrome; T2DM, type 2 diabetes mellitus; AI, adrenal insufficiency; DI, diabetes insipidus; GHD, growth hormone deficiency; HH, hypogonadotropic hypogonadism; DD, developmental delay; PHPT, pseudohypoparathyroidism; AR, autosomal recessive; AD, autosomal dominant; UK, unknown inheritance; and AOM, anti-obesity medications.
